# Pap smear cell image classification using global MPEG-7 descriptors

**DOI:** 10.1186/1746-1596-8-S1-S38

**Published:** 2013-09-30

**Authors:** Luz H Camargo, Gloria Diaz, Eduardo Romero

**Affiliations:** 1 Faculty of Engineering – Cra. 7 No.40 53, Universidad Distrital Francisco José de Caldas, Bogotá D. C. – Colombia; 2 Faculty of Engineering – Cra. 5 No.21 38, Universidad Central, Bogotá D. C. – Colombia; 3 Bioingenium, National University of Colombia, Cra 30 No 45 03-Ciudad Universitaria, Faculty of Medicine - Building 471, National University of Colombia, Bogotá DC - Colombia

## Background

Several strategies have been previously applied for classifying cervical cytology cells, all pursuing a nucleus segmentation. Sanchez sets regions [1] using a simple threshold [2], a procedure broadly adapted to different techniques: a local adaptive segmentation nuclei procedure [3], seed growing [4], mathematical morphology [5], a Hough transform [6], and active contours [7]. Jantzen and Dounias propose several cell features as morphometric descriptors, including the nucleus and cytoplasm areas, nucleus / cytoplasm proportion, nucleus and cytoplasm brightnesses, smaller and larger nucleus/cytoplasm diameters, nucleus and cytoplasm roundness, nucleus and cytoplasm perimeters, nucleus position, nucleus/cytoplasm maxima and minima. Nevertheless, these morphometric characteristics require a previous accurate segmentation, hardly achieved by human intervention using commercial software such as CHAMP (Cytology and Histology Modular Analysis Package, Aarhus, Denmark) or DIMAC (Digital Image Company) [8,9].

## Methods

Rather than attempting to detect some of the previously reported morphometric features, the present investigation used two global MPEG-7 color descriptors, *Color Layout* and *Scalable Color*, and one texture descriptor, the *Edge Histogram descriptor*, as the representation space and two supervised classification algorithms (SVM and KNN) that divide the different classes.

### Classification based on global MPEG-7 descriptors

The cell classification approach is carried out using color and texture MPEG-7 descriptors, thereby attempting to capture information related with the particular color spatial location and global color distribution of both the nucleus and cytoplasm. The texture descriptor stands for the particular borders of both nucleus and cytoplasm and their intrinsic relationships. These global characteristics are not evaluating the classical morphometric features, but they are using nucleus and cytoplasm visual primitives as discriminant factors.

### Color layout

This descriptor, typically used in the YCrCb color space, captures the spatial color distribution in an image or an arbitrary region. Basically, the color layout descriptor uses representative colors on an grid, followed by a Discrete Cosine Transform (DCT) and an encoding of the resulting coefficients. The feature extraction process consists of two parts; grid based representative color selection and DCT transform with quantization. Specifically, an input image is divided into 64 blocks, their average colors are derived and transformed into a series of coefficients by performing a conventional DCT. A few low-frequency coefficients are selected using zigzag scanning and quantized to form a Color Layout Descriptor [10].

### Scalable color

This descriptor is extracted from a color image histogram in the hue-saturation-value (HSV) color space. This histogram, constructed with fixed color space quantization, is projected into a set of Haar bases so that the obtained coefficients constitute a scalable color representation. The histogram values must be normalized and non linearly mapped into a 4-bit integer representation, giving higher weight to small values. The Haar transform is applied then to this histogram version with two basic operators: sum and difference bin neighbor, decomposing the histogram into low and high frequency subbands [10].

### Edge histogram

This descriptor captures the spatial edge distribution, a very useful feature for image matching, even though the underlying texture may not be homogeneous. A given image is first sub-divided into sub-images and local edge histograms, for each of these sub-images, are computed. Edges are then coarsely grouped into five categories: vertical, horizontal, 45 diagonal, 135 diagonal, and isotropic (nonorientation specific). Thus, each local histogram has five bins corresponding to the above five categories. The image partitioned into 16 sub-images results in 80 bins. These bins are nonuniformly quantized using 3 bits/bin, resulting in a descriptor with size of 240 bits [10].

### Classification models

The classification method used a classical K-Nearest Neighbor algorithm and a Support Vector Machine.. The proposed approach was evaluated under a 10-fold experimental setup.

### The k-NN decision rule

The k-nearest neighbor method is an intuitive method that classifies unlabeled samples based on their similarity with samples in the training set. Given the knowledge of N prototype features (vectors of dimension ∑) and their correct classification into M classes, the k-NN rule assigns an unclassified pattern to the class that is most heavily represented among its k neighbors in the pattern space, under some appropriate metric. In this work euclidean distance was used.

### The SVM algorithm

A support vector machine (SVM) is a classification model that finds an optimal separating hyperplane that discriminates two classes. A SVM is a linear discriminator, however it can perform non-linear discriminations thanks to the fact that this is a kernel method. In this work, it is used a SVM version that uses sequential minimal optimization algorithm. The multi-class classification problem is solved using a one vs. all strategy: a binary classifier for each class by labeling the class samples as positive examples and other class samples as negative ones. The final decision is set to the class having the largest decision function among all classes.

## Results and discussion

### Database

Two databases composed of images with single cells, from the Herlev University Hospital, Denmark, were used (http://labs.fme.aegean.gr/decision/downloads). Skilled cyto-technicians and doctors manually classified each cell in 2 classes: abnormal and normal and then subclassified into seven classes. Each cell was examined by two cyto-technicians, and difficult samples also by a doctor. In case of disagreement, the sample was simply discarded (Byriel, Martin, Norup, Jantzen). Finally there are two database:

B_1 data contains 500 cells with the following distribution:

1. Normal: columnar epithelial, parabasal squamous epithelial, intermediate squamous epithelial, superficial squamous epithelial.

2. Abnormal: mild squamous non-keratinizing dysplasia, moderate squamous non-keratinizing dysplasia, severe squamous non-keratinizing dysplasia.

The B_2 data contains 917 cells with the following distribution:

1. Normal: superficial squamous epithelial, intermediate squamous epithelial, columnar epithelial..

2. Abnormal: mild squamous non-keratinizing dysplasia, moderate squamous non-keratinizing dysplasia, severe squamous non-keratinizing dysplasia, squamous cell carcinoma in situ intermediate.

### Experimental Setup

Two classification algorithms were assessed (KNN and SVM). Each classification model was tuned independently for its own particular set of parameters as follows: k-NN was assessed by varying the k nearest neighbors between 1 and 15 with increment steps of two. SVM used two kernel types were evaluated; radial basis function (RBF) and polynomial functions. For the RBF kernel, the γ parameter was varied from 0.00 to 0.90 with increment steps of 0.10, while the polynomial kernel degree was set at 1, 2 and 3. The regularization parameter Complexity 1.0. Evaluation was carried out with both 2-class (normal and anormal) problems. A conventional 10-fold cross validation was performed for every parameter combination.

### Results

**Figure 1:**
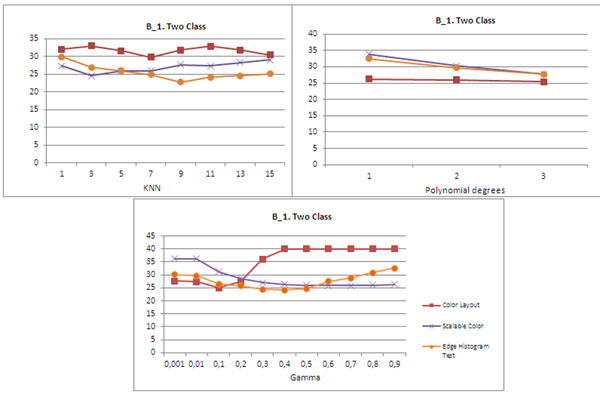
Error for different methods in B_1

**Figure 2:**
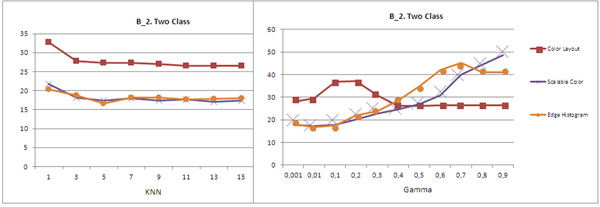
Error for different methods in B_2

The effects of different levels of complexity were evaluated, but do not show important variations. Figure 1 shows the percentage error for the method proposed in the 2- class problem for B_1 datasets. Better performance was achieved by the KNN classifier with k 15. The B_2 database shows the performance of SVM with radial base kernel, for values of 0.01 (Figure 2). In both database these results were obtained with the edge histogram descriptor.

### Discussion

A new strategy for assisting the diagnosis of pap smears which requires no segmentation was proposed, implemented and evaluated. Instead of attempting to segment cells and its structural components, we propose to characterize the internal cell structure using well known MPEG-7 global descriptors. Results are very promising, however evaluation of other specific features and classification algorithms can improve the classification performance.

## Conclusions

This work presented an original method for discrimination of each class: normal and abnormal. Future work is to evaluate the classification of the seven classes.

## Competing interests

The authors declare that they have no competing interests.

## Authors' contributions

All authors read and approved the final manuscript.
